# Probing protein stability: towards a computational atomistic, reliable, affordable, and improvable model

**DOI:** 10.3389/fmolb.2023.1122269

**Published:** 2023-06-01

**Authors:** Germano Nobili, Simone Botticelli, Giovanni La Penna, Silvia Morante, Giancarlo Rossi, Gaetano Salina

**Affiliations:** ^1^ Dipartimento di Fisica, Universitá di Roma Tor Vergata, Roma, Italy; ^2^ INFN, Sezione di Roma Tor Vergata, Roma, Italy; ^3^ CNR—Istituto di Chimica Dei Composti Organometallici, Firenze, Italy; ^4^ Museo Storico della Fisica e Centro Studi e Ricerche E. Fermi, Roma, Italy

**Keywords:** protein stability, protein variant, free energy, unfolding, metastatistics

## Abstract

We present an improved application of a recently proposed computational method designed to evaluate the change of free energy as a function of the average value of a suitably chosen collective variable in proteins. The method is based on a full atomistic description of the protein and its environment. The goal is to understand how the protein melting temperature changes upon single-point mutations, because the sign of the temperature variation will allow us to discriminate stabilizing vs. destabilizing mutations in protein sequences. In this refined application the method is based on altruistic well-tempered metadynamics, a variant of multiple-walkers metadynamics. The resulting metastatistics is then modulated by the maximal constrained entropy principle. The latter turns out to be especially helpful in free-energy calculations as it is able to alleviate the severe limitations of metadynamics in properly sampling folded and unfolded configurations. In this work we apply the computational strategy outlined above in the case of the bovine pancreatic trypsin inhibitor, a well-studied small protein, which is a reference for computer simulations since decades. We compute the variation of the melting temperature characterizing the folding-unfolding process between the wild-type protein and two of its single-point mutations that are seen to have opposite effect on the free energy changes. The same approach is used for free energy difference calculations between a truncated form of frataxin and a set of five of its variants. Simulation data are compared to *in vitro* experiments. In all cases the sign of the change of melting temperature is reproduced, under the further approximation of using an empirical effective mean-field to average out protein-solvent interactions.

## 1 Introduction

Many proteins are characterized by a given three-dimensional structure when they are observed in a water soluble monomeric state Branden and Tooze (1999). In order to understand the way the sequence determines the structure, the effect of single point mutations has been studied since a long time Cunningham and Wells (1989). A simple way to address the sequence-structure interplay is to measure some structural parameter as a function of temperature. Circular dichroism (CD) and many other techniques are often used and in many cases the change of this structural parameter with temperature can be taken as an indicator of the melting of the protein structure Cantor and Schimmel (1980). The change of melting temperature triggered by different single point mutations is therefore a widely used measure of the change of protein stability upon a localized change of the protein sequence and large archives of such data have been collected Guerois et al. (2002); Alexov and Sternberg (2013); Forbes et al. (2016). When this information is available, it must be interpreted in terms of the reshaping of atomic interactions.

The change of protein stability upon sequence mutations has implications in many pathologies. One example is Friedreich’s ataxia, an autosomal-recessive genetic condition that causes ataxia, sensory loss and cardiomyopathy worsening over time Pandolfo (2009); Klockgether (2011); Clark et al. (2018). The cause of the disease is in mutations of the gene encoding for the frataxin (FXN) protein. Depending on the specific kind of mutation, a patient may end up with an insufficient level of frataxin, a nonfunctional frataxin or frataxin, that is, not correctly localized in the mitochondria Delatycki et al. (2000); Galea et al. (2016). Frataxin variants have also a role in cancer, as expected because of the involvement of FXN and mitochondria in the control of oxidative metabolism Schulz et al. (2006). Indeed, missense variants are found in multiple human cancer tissues Petrosino et al. (2019, 2021). The example of FXN shows that even single point mutations can have significant impact in protein stability, trafficking, plasticity, interactions with local environment and mutual interactions with other macromolecules.

Many models have been proposed to relate measured changes in protein stability with the chemical nature of the protein sequence. High through-put approaches based on atomic models have been recently developed Steinbrecher et al. (2017). Many of these approaches are summarized in this Special Issue.

The method we would like to propose here aims at predicting the change of thermal stability of a protein in a monomeric water-soluble state, when its sequence is changed by a single aminoacid. The method is based on a suitable modelling of interatomic forces, i.e., it is atomistic, and includes an explicit model of the water solution. The method was initially applied to FXN Botticelli et al. (2022) and it is here refined and discussed in more detail, to achieve better computational performance and higher accuracy in prediction. In particular, we use here the well-tempered metadynamics, one of the best performing method to enhance sampling of phase space in atomistic models. A small reference protein of 58 residues is first used to assess the methods and to understand limitations and advantages.

Many initial configurations of the protein of interest are generated, assuming the protein structure representing the native state of the wild-type sequence, but with initial conditions diversified as much as possible. This is achieved by changing the protein environment, that is, in this case the water solution of NaCl. A multiple walkers metadynamics simulation is carried out Raiteri et al. (2006); Hošek et al. (2016); Hošek et al. (2017), building an external biasing potential as a function of a suitably chosen collective variable. In the range of values spanned by the collective variable folded and unfolded protein structures are sampled.

The external potential is built so as to initially unfold the protein structure. All along the metadynamics simulation time, the external biasing potential is systematically constructed and updated to uniformly sample folded and unfolded configurations. This goal is best achieved by combining multiple walkers histories into a unique trajectory Hošek et al. (2017).

The standard analysis of multiple walkers metadynamics can be performed, but limitations in predicting experimental behaviours arise because of the huge number of configurations required to achieve a good convergence and stability. We therefore decided to exploit the features of the maximal constrained entropy approach which allows to properly re-modulate the collected set of sampled configurations by imposing suitable conformational constraints. In this way we can reliably monitor the change in free energy as a function of average values of the chosen collective variables. The quantity of interest to be calculated is therefore:

$$\Delta \Delta G=\Delta G_{X}-\Delta G_{0}=\left[G_{X}\left(s\right)-G_{X}\left(s_{0}\right)\right]-\left[G_{0}\left(s\right)-G_{0}\left(s_{0}\right)\right],$$ (1)

where the subscript denotes a sequence (the pedix 0 indicates a reference sequence, usually the wild-type), and the *s* state variable indicates the degree of structural order, where *s*_0_ indicates the folded native state and *s* indicates the unfolded state.

The approach is first applied to the study of a paradigmatic protein displaying a well defined three-dimensional structure, namely, to the bovine pancreatic trypsine inhibitor (BPTI), where the effect of a set of mutations on melting temperature has been carefully investigated Yu et al. (1995). BPTI has always been a milestone for folding studies, being one of the smallest proteins (58 aminoacids) characterized by a well defined structure. Then, the same procedure is applied to frataxin, in a truncated form of 121 aminoacids, and 5 of its variants Petrosino et al. (2019).

## 2 Materials and methods

The computational methods used in this work are similar to those used in Ref. (Botticelli et al., 2022). In the following we emphasize the differences that characterize this work.

### 2.1 Metadynamics

Let *ξ*(*q*) be a collective variable (CV) function of atomic positions, *q*. When *ξ* is an observable quantity, the values, *s*, allowed for *ξ* can be used to label system macrostates. The set of coordinates *q* labels the system microstates, each set of *q* yielding one of the possible values of *s*. If ergodicity holds, infinitely long simulations of a trajectory *q*(*t*) in a given statistical ensemble would correctly sample the statistical weight of *ξ*. However, because of the huge number of ways in which certain values of *s* of *ξ* are encountered, compared to others, actual numerical simulations in practice only sample the maximally degenerate values of *ξ*. This is precisely the case where *ξ* is the CV associated to folding/unfolding events.

Standard statistical ensembles and more recently generalized ensembles try to address this problem by biasing the trajectory to spend more time where *ξ* has a low degeneracy and less time where *ξ* has a large degeneracy.

The sampling of configurations obtained with the biased inverse probability of *ξ* is called metastatistics. We will denote by $\tilde{P}\left(q\right)$ the probability of microstates encountered along the simulated trajectory and by $\tilde{P}\left(\xi \right)$ the probability of the macrostates labeled by *ξ*. For simplicity with a little abuse of notation we use the same name for the metastatistics probability as function of the microscopic variables, *q*, and to the associated metastatistics probability as function of the macroscopic collective variable, *ξ*.

Many methods have been proposed to sample configurations with the inverse of the estimated probability of *ξ* Mitsutake et al. (2001). In this work and in the previous application of the method Botticelli et al. (2022), we used the altruistic metadynamics proposed in Refs. Hošek et al. (2016); Hošek et al. (2017). The desired metastatistics is obtained from a swarm of trajectories provided by metadynamics after building a suitable external bias, which is then kept fixed when collecting configurations in the final step of the *NpT* simulation (see Section 2.6). We performed simulations in the statistical ensemble associated to constant temperature *T* and pressure *p* (*NpT* ensemble) because macromolecules forced by an external bias undergo large and fast conformational changes. When these conditions occur, solute macromolecules exert strong perturbations over the explicit solvent and ions representing their environment. To cope with steep changes of kinetic energy of water molecules and possible temporary voids around the macromolecule, the *NpT* ensemble is recommended.

In the framework of metadynamics, the estimated probability of the CV is expressed by means of a sum of Gaussian functions, *V*_*G*_ [*ξ*(*q*)], related to the inverse metastatistics probability by the formula

$$\ln\tilde{P}\left(\xi \right)=\beta V_{G}\left[\xi \left(q\right)\right]+C,$$ (2)

with *β* = 1/(*k*_*B*_ *T*) where *T* is the temperature used in the simulation, *k*_*B*_ the Boltzmann constant, and *C* a normalization constant, that is, of no relevance in the computation of thermal averages. Different methods have been proposed to build an external bias *V*_*G*_ [*ξ*(*q*)] such that the probability distribution of *ξ* is flat and transitions between folded and unfolded states of a biomolecule endowed with many degrees of freedom, are equally well sampled.

In metadynamics the external potential *V*_*G*_ (*ξ*, *t*) acting on the system at time t is defined as:

$$V_{G}\left(\xi \left(q\right),t\right)=w\underset{t^{\prime }=\tau_{G},2\tau_{G},\ldots }{\sum }\exp\left(\frac{-{\left(\xi \left(q\right)-s\left(t^{\prime }\right)\right)}^{2}}{2\delta^{2}}\right)$$ (3)

where *t*′ < *t*, *s*(*t*) = *ξ*(*q*(*t*)) is the value taken by the CV at time *t*, *w* is the Gaussian height, *δ* is the Gaussian width *τ*_*G*_ is the time interval after which a new Gaussians is added.

After a sufficiently long time *V*_*G*_(*s*, *t*) provides an estimate of the underlying free energy *F* according to the formula

$$V_{G}\left(s,t\right)=-F\left(s\right)+C\left(t\right)$$ (4)

where *C*(*t*) depends on time but not on the collective variables *s*, *V*_*G*_ is the external biasing potential acting on the system at time *t*.

Equation above states that an equilibrium quantity, like free energy, can be estimated by a non-equilibrium dynamics in which the bias potential is changed in time, as new Gaussians are successively added. In metadynamics, when all the wells in CV distribution are filled with Gaussians, the dynamics in the CV space becomes diffusive.

### 2.2 Well tempered metadynamics

Well tempered metadynamics is an improved approach designed to obtain a reliable estimator of the free energy Barducci et al. (2008). The weight of each Gaussian function added to the bias *V*_*G*_ depends on the history of *V*_*G*_ (*V*_*G*_(*t*′)). Equation 3 changes into:

$$V_{G}\left(\xi \left(q\right),t\right)=w\underset{t^{\prime }=\tau_{G},2\tau_{G},\ldots }{\sum }\exp\left(\frac{-V_{G}\left(\xi \left(q\right),t^{\prime }\right)}{k_{B}\Delta T}\right)\times \exp\left(\frac{-{\left(\xi \left(q\right)-s\left(t^{\prime }\right)\right)}^{2}}{2\delta^{2}}\right)\;,$$ (5)

where *k*_*B*_Δ*T* is approximately the energy change when a new value of *ξ* is visited. An exact relation between *V*_*G*_(*s*, *t*) and *F*(*s*) can be obtained if the rate at which the bias potential is modified is suitably decreased as the simulation progresses. With well tempered metadynamics, the biasing potential converges to

$$V_{G}\left(s,t\right)=-\frac{\Delta T}{T+\Delta T}F\left(s\right)+C\left(t\right).$$ (6)

The quantity $T+\frac{\Delta T}{T}$ is called “biasing factor”.

For a finite *T*, the probability distribution is proportional to:

$$\exp\left(\frac{-F\left(s\right)}{k_{B}T}\right)\exp\left(\frac{\Delta T}{T+\Delta T}\frac{F\left(s\right)}{k_{B}T}\right)=\exp\left(-\frac{F\left(s\right)}{k_{B}\left(T+\Delta T\right)}\right)$$ (7)

which corresponds to effectively increasing the CV sampling temperature. Thus, the effect of well tempered metadynamics is similar to that of other non-equilibrium methods, like steered molecular dynamics, but trajectories are obtained with a quasi-equilibrium procedure Bussi et al. (2018).

In well tempered metadynamics, as the simulation proceeds the width of the added Gaussian remains constant but its height decreases (see Eq. 5). The bias, which increases monotonically, eventually changes very slowly with time. At the beginning the space of CV is flooded by Gaussians of height *w*. With the progress of flooding, heights of newly added Gaussians decrease. This behaviour is very important in highly complex biological systems, where the bias potential should never reach any excessively large value.

In contrast with the “non tempered” metadynamics, in the well tempered metadynamics a flat CV distibution is not expected to be achieved when convergence is obtained. A simple interpretation of the fact that the distribution of the CV at long times is not flat is the following. Since the prefactor for the accumulated Gaussians depends on the value of *s*, Gaussians of different heights are placed in different regions of the CV space. In order to reach a stationary distribution, it is thus necessary that the system spends more time in regions where small Gaussians are added and less time in regions where large Gaussians are added. This idea can be pushed further and used to convert metadynamics in an algorithm, not designed to flatten completely (as in non-tempered metadynamics) or partially (as in well tempered metadynamics) the histogram of the CVs but rather to enforce a predefined distribution Bussi et al. (2018).

In this work we used a biasing factor of 20 (see Eq. 6), corresponding to Δ*T* = 5700 K, in agreement with the biasing factor used in literature for similar molecular systems Hošek et al. (2017). The energy value *R*Δ*T* is of the order of a typical energy barrier of a single hydrogen bond.

### 2.3 The maximal constrained entropy method

The maximal constrained entropy method (MEC method, hereafter) allows, starting from $\tilde{P}\left(\xi \right)$ of Eq. 2, to obtain a better probability for thermal average calculations. This elaboration is used to correct for limitations of $\tilde{P}\left(\xi \right)$ , whatever the method used for its determination is. We remind that the method consists in post-processing the biased statistics (that we indicate with metastatistics) collected by whatever method. Since in actual simulations one works with trajectories where configurations can be enumerated, we attach the microstate index *γ* to the configuration {*q*} and we denote by ${\tilde{P}}_{\gamma }$ the probability

$${\tilde{P}}_{\gamma }=\frac{{\tilde{w}}_{\gamma }}{\sum_{\gamma }{\tilde{w}}_{\gamma }},$$ (8)

where ${\tilde{w}}_{\gamma }$ is the number of microstates with label *γ* collected in the metastatistics and $\tilde{Z}=\sum_{\gamma }{\tilde{w}}_{\gamma }$ is a normalization factor.

In an infinitely long (ergodic) simulation, it is unnecessary to explicitly evaluate the weights ${\tilde{w}}_{\gamma }$ , as they are automatically encoded in the degeneracy of the set of collected configurations sampled along the simulated trajectory. This means that in the following equations, where the sum over *γ* is extended over that actually produced configurations, we should not include the factor ${\tilde{P}}_{\gamma }$ . However, we leave this redundant factor to recall that we are dealing with a finite set of configurations generated by metadynamics.

In case of the “non tempered” metadynamics, the maximal constrained entropy was employed as a viable solution to compute thermal averages as a function of the average values taken by the chosen CV, in situations where metastatistics is not fully ergodic and the CV distribution does not come out flat. As mentioned, in the case of well tempered metadynamics the CV distribution is not expected to be flat, but the maximal constrained entropy method is a powerful method to “correct” the free energy by adding *ex post* further information about the system injecting extra information. In our case we use the maximal constrained entropy to introduce in the computation of the free energy the change of number of hydrogen bonds in *α*-helices in folding↔unfolding processes. In general the maximal constrained entropy method can be used either to improve the estimate of the free energy for a non-converging system (e.g., in a metadynamics simulation the CV distribution is not flat) or to compute the free energy by reintroducing *ex post* degrees of freedom related to the CV (like the *α*-helices’ hydrogen bonds in the case of frataxin, see Section 3). This second use of the maximal constrained entropy method is really powerful because allows to have a reliable estimate of the free energy while keeping efficient the simulations by limiting the degrees of freedom of the system.

### 2.4 Estimating the free energy

The main goal of this work is to compare the change of free energy as a function of the number of hydrogen bonds (*s*) computed using well tempered metadynamics and maximal constrained entropy, with the results obtained in protein thermal denaturation experiments Yu et al. (1995); Petrosino et al. (2019). Both BPTI and FXN are folded in a structure where one or two *α*-helices lay over a small *β*-sheet. The experimental measurement of the free energy difference between folded and unfolded states was obtained by measuring the molar ellipticity at 222 nm, a wavelength where the contribution of *α*-helix to CD spectra is dominant. Besides acting on the *α*-helices arrangement, the protein ternary structure can also be perturbed by destroying the intra-molecular hydrogen bonds that stabilize the *β*-sheet. For a small protein like BPTI (58 residues), we decided to include in the CV all the hydrogen bonds that are formed in the native folded state Parkin et al. (1996). For FXN (121 residues) we took instead as a CV the number of hydrogen bonds occurring in the *β*-sheet formed by 4 anti-parallel *β*-strands, which are observed both in 1EKG and 5KZ5 structures Botticelli et al. (2022). This choice in the case of FXN was made to reduce the number of degrees of freedom of the system thus substantially decreasing the time required to sample its phase space. The use of such CV as a way to monitor the structural transitions in the protein was inspired by several previous applications of metadynamics Barducci et al. (2006).

For both proteins and variants, the biasing potential, *V*_*G*_, was obtained at the end of a systematic construction (well tempered metadynamics) in which *V*_*G*_ is progressively built by summing over Gaussian functions of the CV. Gaussian functions (possibly scaled by the biasing factor in the case of the well tempered metadynamics) are deposited every 20 ps along the molecular dynamics (MD) simulation time.

The accumulated final biasing potential, *V*_*G*_(*ξ*), smoothly interpolated by a polynomial of fourth order, was used for the direct computation of the change in the free energy for folded to unfolded states and *vice versa*. The free energy change defined in well tempered metadynamics is given by Eq. 6:

$$F\left(s\right)-F\left(s_{0}\right)=-\left(\frac{T+\Delta T}{\Delta T}\left[V_{G}\left(s\right)-V_{G}\left(s_{0}\right)\right]\right)$$ (9)

with *s*_0_ a reference state corresponding to a given value of the CV and *V*_*G*_ the external biasing potential determined at the end of construction. Equation 9 holds also in the *NpT* statistical ensemble, when the construction of the bias *V*_*G*_ is performed in such statistical ensemble. In this case, the Helmoltz free energy *F*(*s*) is replaced by the Gibbs free energy *G*(*s*). We call the latter function *G* free energy, hereafter, for simplicity. The *G* free energy extracted from well tempered metadynamics simulations (Eq. 9), was then compared with the *G* free energy obtained with the maximal constrained entropy method.

The accumulated statistics used in the successive maximal constrained entropy application have been obtained by collecting the system configurations along a trajectory where the biasing potential was kept fixed (i.e., not anymore updated). Within the maximal constrained entropy method, the definition of the *G* free energy (see La Penna et al., 2004) is given by the formula

$$G\left(s\right)={\left\langle H\right\rangle }_{\lambda }-T\;k_{B}\;{\bar{S}}_{c}\left(s\right),$$ (10)

in which *G*(*s*) is written as the combined sum of the enthalpy in the *NpT* ensemble, and the (informational) entropy measured by the maximal cross-entropy. In Eq. 10 *H* = *U* + *pV* is the enthalpy of the simulated system, *λ* the parameter associated with the constraint, ${\bar{S}}_{c}$ the maximal cross-entropy change due to the introduction of such a constraint, *k*_*B*_ the Boltzmann constant, and *T* some effective temperature in the stability range of the system under study. The same free energy definition holds for the Helmoltz free energy *F* when the enthalpy *H* is replaced by the energy *U* if one is working in a *NVT* ensemble.

The maximal cross-entropy in Eq. 10 is described in the following. Given an estimate, ${\tilde{P}}_{\gamma }$ , of the metastatistic probability, say the one provided by metadynamics, the problem of finding the least-biased expression of the probabilities *P*_*γ*_, that is, nearer to ${\tilde{P}}_{\gamma }$ and satisfies the condition

$$s=\left\langle \xi \right\rangle =\underset{\gamma }{\sum }P_{\gamma }\xi_{\gamma },$$ (11)

is solved by determining the maximum of the cross-entropy functional Attard (2000); La Penna (2003); La Penna et al. (2004).

$$S_{c}\left[P,\tilde{P}\right]=-\underset{\gamma }{\sum }P_{\gamma }\ln\frac{P_{\gamma }}{{\tilde{P}}_{\gamma }}.$$ (12)

under the constraint (Eq. 11). The well-known solution of this variational problem is given by the formulae:

$$P_{\gamma }=\frac{{\tilde{P}}_{\gamma }}{Z_{\lambda }}\exp\left(-\lambda \xi_{\gamma }\right)$$ (13)

$$Z_{\lambda }=\underset{\gamma }{\sum }{\tilde{P}}_{\gamma }\exp\left(-\lambda \xi_{\gamma }\right)$$ (14)

with the parameter *λ* the solution of the (highly non-linear) equation:

$$s=\underset{\gamma }{\sum }P_{\gamma }\xi_{\gamma }=\frac{1}{Z_{\lambda }}\underset{\gamma }{\sum }{\tilde{P}}_{\gamma }\exp\left(-\lambda \xi_{\gamma }\right)\xi_{\gamma }.$$ (15)

The quantity exp (−*λξ*_*γ*_)/*Z*_*λ*_ is called the modulation factor of the metastatistics. Owing to Eq. 15, *λ* is a function of *s*. Inserting the solution for *P*_*γ*_ into Eq. 12 one gets for the cross entropy at its maximum:

$${\bar{S}}_{c}\left(s\right)=\ln Z_{\lambda }+\lambda \;s.$$ (16)

The average of *H* (or simply of *U* in *NVT* simulations) is obtained using equations like

$$b_{\lambda }={\left\langle B\right\rangle }_{\lambda }=\frac{1}{Z_{\lambda }}\;\underset{\gamma }{\sum }{\tilde{P}}_{\gamma }\exp\left(-\lambda \xi_{\gamma }\right)\;B\left(q_{\gamma }\right),$$ (17)

with *B* either *H* or *U* and $Z_{\lambda }=\sum_{\gamma }{\tilde{P}}_{\gamma }\exp\left(-\lambda \xi_{\gamma }\right)$ . The identification of *S*_*c*_ and *T* in Eq. 10 with, respectively, thermodynamic state function entropy *S* and state variable absolute temperature *T*, is empirical. It must be noticed that changes in thermodynamic *T S* values are also reflected in the changes of ⟨*H*⟩_*λ*_ as a function of *λ*.

The details to compute the free energy within the maximal constrained entropy method, the direct calculation of ⟨*H*⟩_*λ*_ in Eq. 10 as well as the free energy error estimate is the same we used in our previous work where the “non tempered” version of the metadynamics Botticelli et al. (2022) was employed. In this work we concentrate on collecting more accurate averge quantities (well-tempered metadynamics and longer simulations) and on applying the proposed method also to a simpler protein (BPTI). We must note that the direct calculation of ⟨*H*⟩_*λ*_ in Eq. 10 includes the effects of the fluctuations of *U* and *V* due to the movement of all explicit water molecules and ions included in the atomistic model of the protein environment. The fluctuations of *H* are huge, while the change of the average of *H* with *s* is small. This is a serious issue when using the total enthalpy like in Eq. 10. As it is customary done in these cases, we use an approximate evaluation of ⟨*H*⟩_*λ*_, where *H* is replaced by the effective mean-field free energy $\bar{U}$ of the protein solute. The advantage of this approximation is that the energy of the system is thermally averaged over the many degrees of freedom of water molecules and ions surrounding the much smaller solute protein aggregate.

A widely used strategy for the evaluation of the effective mean-field energy of the solute protein is the so-called molecular mechanics/Poisson-Boltzmann solvent accessible approximation (MM/PBSA) Simonson et al. (2002). In this approach the mean-field energy for solute-solvent interactions is described as the sum of polar (electrostatic) and non-polar (surface) contributions. For each protein configuration *Q* one writes

$$\bar{U}\left(Q\right)=U_{intra}\left(Q\right)+G_{solv,np}\left(Q\right)+G_{solv,pol}\left(Q\right),$$ (18)

where *U*_*intra*_ is the intra-molecular part of the potential energy in the protein force-field, given by

$$U_{intra}\left(Q\right)=U_{str}\left(Q\right)+U_{bend}\left(Q\right)+U_{tors}\left(Q\right)+U_{vdw}\left(Q\right)+U_{el}\left(Q\right).$$ (19)

The various contributions are the stretching (*U*_*str*_), bending (*U*_*bend*_), and torsional (*U*_*tors*_) terms in the potential. *U*_*vdw*_ and *U*_*el*_ are the Lennard–Jones and Coulomb interactions, respectively, computed by summing over all the pairs of atoms of the protein.

The last two terms in Eq. 18 represent solute-solvent contributions to free energy at fixed *Q*. Mean field energy is the energy as a function of *Q* once the variables associated to solvent positions and velocities are averaged for the given value of solute positions *Q*. The averaging is performed at the given thermodynamic state variables *p* and *T* used in the simulation of the whole system. Within this mean-field assumption, the solute and the solvent are made independent. This is a strong approximation, since the chosen collective variable contains the number of hydrogen bonds within protein groups and once a single intramolecular hydrogen bond is broken there is a large chance for the formation of hydrogen bonds with the water molecules in the protein environment where the breaking event occurs. On the other hand, this elementary change of free energy, that does not imply a wide change in protein structure, can be calculated within the MM/PBSA approximation as the sum of *G*_*solv*,*np*_ and *G*_*solv*,*pol*_. Therefore, under this approximation, the change of free energy *G*(*s*) depends on the number of protein configurations for which a unitary change of *s* is allowed independently of the configuration of the protein environment. The calculation of *G*_*solv*,*np*_ and *G*_*solv*,*pol*_ is described in the following.

The term *G*_*solv*,*np*_ is the contribution to the solute-solvent free energy due to the formation of a cavity of zero charge density with the shape of the solute protein and the creation of the solute-solvent interface. Introducing a charge density in the space occupied by the solute leads to the *G*_*solv*,*pol*_ contribution. The charge density is given in terms of the point charges *q*_*i*_ of the atom *i* sitting at the point ${\vec{r}}_{i}$ , where *i* runs over the *N*_*a*_ atoms of the solute molecule.

The term *G*_*solv*,*np*_ is calculated as an empirical linear combination of the solvent accessible surface area (SASA) for each group in the solute molecule Ooi et al. (1987) according to the formula

$$G_{solv,np}=\overset{N_{a}}{\underset{i}{\sum }}\sigma_{i}{SASA}_{i},$$ (20)

where the coefficients *σ*_*i*_ are positive or negative for hydrophobic or hydrophilic groups, respectively (see below for details). Finally the electrostatic contribution to the solute-solvent free energy, *G*_*solv*,*pol*_, is given by the electrostatic energy required to charge the low-dielectric solute molecule of generic shape into a high-dielectric medium like a salt-water solution. The magnitude of this contribution is obtained by a numerical finite difference solution of the Poisson–Boltzmann equation Rocchia et al. (2002).

### 2.5 Summary of the method

We summarize the complicated computational protocol of our theoretical analysis as follows. One starts by performing MD simulations at *T* = 300 K in the presence of the biasing potential *V*_*G*_(*ξ*) built according to the well tempered metadynamics strategy. The resulting statistics is what we call metastatistics. Using the set of collected configurations, we determine the *λ* parameter that maximizes the cross-entropy *S*_*c*_ in the maximal constrained entropy method, under the constraint ⟨*ξ*⟩ = *s*. In the case of BPTI, *ξ* is the number of hydrogen bonds holding together the protein *α*-helices and *β*-sheet secondary motifs. In this case, the *ξ* of metadynamics and that of maximal constrained entropy coincide. In the case of BPTI, differently from FXN (see below), the *ξ* collective variable takes into account the whole of the secondary structure as it is observed in the crystal folded structure. Therefore, *s* takes integer values in the range between 0 and 16. In the case of FXN, the variable *ξ* used in metadynamics is the number of hydrogen bonds holding together the protein *β*-sheet (made of 4 anti-parallel *β* strands). The values of *s* are in the range between 0 and 15. But in the maximal constrained entropy approach we extended *ξ* adding to it the number of hydrogen bonds in the two *α*-helices. For each value of *s*, we get a value of *λ* that yields the modulating weight

$$w\left[q\left(t\right)\right]=\frac{1}{Z_{\lambda }}\;\exp\left\{-\lambda \xi \left[q\left(t\right)\right]\right\},$$ (21)

with *q* the system configuration at time *t*, indexing the microstate *γ*, along the collected metadynamics trajectory. For details see Ref. (Botticelli et al., 2022).

### 2.6 Simulation parameters

Apart from the fact that differently than what was done in Ref. (Botticelli et al., 2022), in this work the metastatistics is obtained as altruistic multiple-walkers well-tempered metadynamics, most of the technical details of the simulation procedure we followed to compute the expectation values of the physical quantities of interest described in Section 2 are identical to those reported for FXN in Ref. (Botticelli et al., 2022). Below we only outline the few differences.

Table 1 provides the list of hydrogen bonds used to define the CV for the BPTI. All the hydrogen bonds contribute to the BPTI CV and are used both in well tempered metadynamics and maximal constrained entropy. For FXN only the number of hydrogen bonds in the *β*-sheet, *β*_1−4_, is used to generate the statistics of metadynamics. However, the total number of hydrogen bonds listed in Table 1 of Ref. (Botticelli et al., 2022), including the two *α*-helices added to the definition of CV in the successive maximal constrained entropy step. We call this an extension of the CV *ξ* used in metadynamics and we indicate it with *ξ*′. The corresponding constrained average is indicated with *s*′.

**TABLE 1:** Pairs of atoms used in Eqs 16–18 of Ref. (Botticelli et al., 2022) and related label in parameter *S*. As for FXN, see Table 1 of the same publication. Residues are those of BPTI WT sequence. Mutated residues are boldface.

*β*_1−2_		*α*_1_		*α*_2_	
N (Tyr 35)	O (Ile 18)	N (Cys 5)	O (Pro 2)	N (Met 52)	O (Ala 48)
N (Ile 18)	O (Tyr 35)	N (Leu 6)	O (**Asp 3**)	N (Arg 53)	O (Glu 49)
N (Phe 33)	O (Arg 20)	N (Glu 7)	O (**Phe 4**)	N (Thr 54)	O (Asp 50)
N (Arg 20)	O (Phe 33)			N (Thr 54)	O (Ala 51)
N (Gln 31)	O (Phe 22)			N (Cys 55)	O (Ala 51)
N (Phe 22)	O (Gln 31)				
N (Leu 29)	O (Asn 24)				
N (Asn 24)	O (Leu 29)				

Table 2 reports the number of atoms of the two systems (BPTI and FXN) we have studied. In the case of BPTI, the structure of the unique folded structure available [1BPI PDB entry Parkin et al. (1996)] has been used. As for FXN, the initial configurations of the various walkers are obtained using the available crystallographic information about the native FXN protein sequence. We used two structures: the X-ray structure of the mature human frataxin [PDB 1EKG, segment 88-210 Dhe-Paganon et al. (2000)]; the structure of FXN in the mitochondrial iron-sulfur cluster assembly machine as it was determined by electron microscopy (PDB 5KZ5, chain A, segment 42-210 Gakh et al. (2016)).

**TABLE 2:** Short description of the atomistic models used in metadynamics simulations. The composition of each system changes only in the protein sequence for each protein (BPTI and FXN, respectively). The number of water molecules and counterions (NaCl) is the same for all the 90 walkers representing each system, and the same (= symbol) for different variants of the same protein.

System	Protein atoms	Water molecules	Na	Cl
BPTI				
BPTI [5-55]_BPTI_	892	11033	21	27
D3A	890	=	=	=
F4A	890	=	=	=
FXN				
WT	1875	13926	34	26
D104G	1870	=	=	=
A107V	1881	=	=	=
F109L	1874	=	=	=
Y123S	1865	=	=	=
S161I	1883	=	=	=
W173C	1862	=	=	=
S181F	1884	=	=	=
S202F	1884	=	=	=

The values of *α* and *w* of Eq. 3 in Ref. (Hošek et al., 2017) and used in the successive stages of the simulation are reported in Table 3. As for the construction of the biasing potential, we remark that its construction in the present work lasted 22 ns, while in our previous application it lasted 16 ns. The exchange of the bias among walkers takes place every 2 ns. At the end of stage 15 (see Table 3), i.e., after simulating each walker for a total of 30 ns using an altruistically updated bias, the external bias that will be used in stage 16 and in the following steps is not updated any more. From stage 16 to the end the final metastatistics is collected, storing configurations along the simulated trajectory every ps. The time duration of this last simulation step was 10 and 30 ns for BPTI and FXN, respectively.

**TABLE 3:** Short description of the simulation stages used to build the external bias *V*_*G*_(*ξ*) and to acquire the metastatistics at constant external bias. Where *α* and *w* are not indicated, the external bias is not updated. The initial bias is zero. Therefore, stages 1–3 (6 ns) are equilibration stages. The bias construction is the same for all variants of BPTI and FXN. As for the constant bias simulation stage 16–20 (10 ns) were collected for BPTI, while 16-30 (30 ns) were collected for FXN. Values of *α* and *w* are used when applying the altruistic combination of single-walker updating (2 ns) of *V*_*G*_ using Eq. 3 of Ref. (Hošek et al., 2017). The resulting global altruistic bias is used in the following 2-ns stage (next line). The bias after stage 15 is approximately the same for all walkers and, therefore, is made identical for all walkers by averaging over the 90 walkers.

Stage	Time length	*α*	*w*
1–4	8	-	-
5	2	0	1
6	2	1/4	1
7	2	1/2	1
8	2	3/4	1
9–15	14	1	1/2
16-end	10–30	-	-

## 3 Results

### 3.1 Bovine pancreatic trypsin inhibitor (BPTI)

48 single point mutations have been studied in the case of BPTI in the literature Yu et al. (1995) via alanine-scanning. This set of mutations includes all residues, with the exception of 6 Ala and 4 Cys, mutated to Ala. The reference sequence used to study the change in melting temperature is the native sequence where Cys 14, 30, 38, and 51 are mutated in Ala. This reference variant is indicated as [5-55]_BPTI_, to underline the presence of the residual 5–55 disulfide bridge. The sequence is used because the native sequence has 3 disulfide bridges in the folded structure and it does not unfold at *T* < 100°C. The removal of 2 disulfide bridges allows the melting at *T* < 50°C, while the protein keeps the same folded structure as the native (WT) sequence, as summarized in Ref. (Yu et al., 1995). Therefore, we could use the structure determined for the WT sequence as the initial representation of the folded state (1BPI Parkin et al. (1996) in PDB).

According to our conventions, a positive ΔΔ*G* means a larger reversible work required to unfold the given variant with respect to the reference sequence. All the variants analyzed in experiments have been already studied as part of large data-sets in previous works dedicated to predictions of free energy change Guerois et al. (2002); Steinbrecher et al. (2017). In our work we are interested in predicting the sign of the free energy change, which is also the sign of the change of the melting temperature *T*_*m*_, Δ*T*_*m*_. As paradigmatic cases we focused, among the 48 variants, on the two displaying the largest measurable change in the absolute value of Δ*T*_*m*_. The mutations with the most positive and negative value of Δ*T*_*m*_ [see Table 1 in Ref. (Yu et al., 1995)] are D3A and F4A, respectively.

**FIGURE 1:**
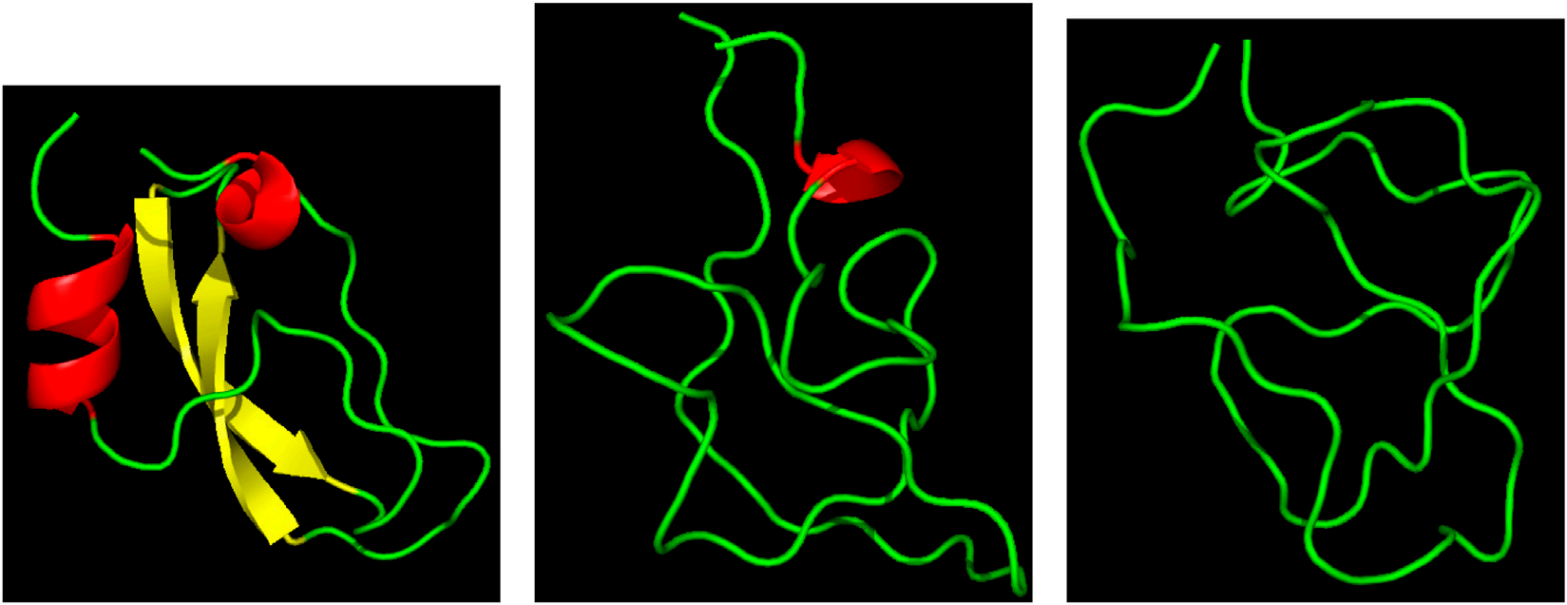
Three representative structures of [5-55]_BPTI_ reference sequence of BPTI. Left—*ξ* = 13 (folded state); middle—*ξ* = 4 (unfolded state); right—*ξ* = 4 (unfolded state). *α*-helices are in red; *β*-sheet is in yellow; the displayed ribbon interpolates the backbone atoms. The Pymol program is used for the molecular drawing Schrödinger (2015).

Three representative structures of the [5-55]_BPTI_ reference sequence of BPTI. are displayed in Figure 1 to show how the folded (left panel) and unfolded (right panel) states look like in terms of atomic configurations. Native BPTI is folded into a ternary structure with two short *α*-helices and a small *β*-sheet. The construction of the external bias, *V*_*G*_(*ξ*), perturbs the ternary structure by breaking the intramolecular hydrogen bonds.

This is why we decided to take as a collective variable *ξ* the sum of the number of hydrogen bonds between the two *α*-helices (*α*) and the *β*-sheet (*β*). The number of hydrogen bonds of *α*-helices and *β*-sheets in the initially folded structure (PDB 1BPI) is 8 for both secondary structures. Therefore, the values of *ξ* span the range between 0 and 16. Figure 1 shows in the right panel that the unfolded state is represented by a molten globule. This occurs because of the short-range nature of the collective variable we have chosen. In the specific case of BPTI the presence of the residual disulfide bridge 5-55 that seals the N-terminus with the C-terminus also pushes the protein towards this atomic arrangement.

**FIGURE 2:**
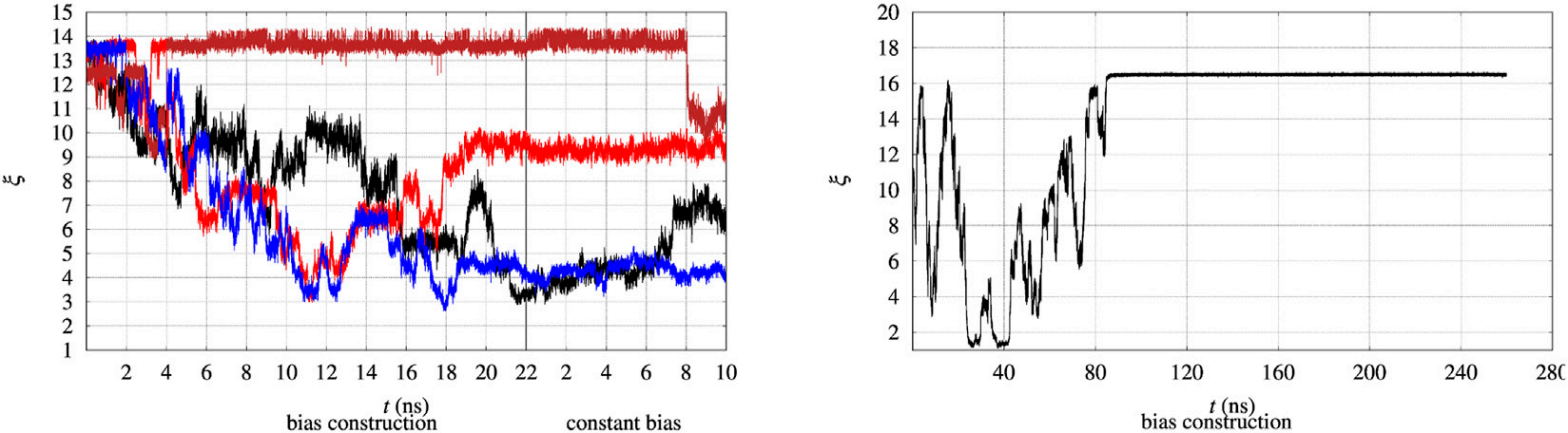
Time evolution of the collective variable *ξ* during the bias construction (left part) and at constant bias (right part), with the vertical line dividing the bias construction from the bias application. Left—The evolution is displayed in different colors for 4 representative walkers among 90 and for BPTI in the [5-55]_BPTI_ sequence. Right—The same evolution is displayed for a single walker in well-tempered metadynamics of FXN in the WT sequence.

The evolution in time of the collective variable *ξ* is notoriously slow, even by using well tempered metadynamics. Therefore the convergence of the external bias *V*_*G*_(*ξ*) is expected to occur after very long simulation times. This issue is illustrated in Figure 2, where the time evolution of *ξ* of 4 walkers among 90 is displayed. We remind that every 2 ns the bias *V*_*G*_ obtained by the whole set of 90 walkers is exchanged among all of the walkers during bias construction in the altruistic approach [Eq. 3 in Hošek et al. (2017)]. Furthermore, before the bias construction the 90 walkers have been separately equilibrated for 8 ns. The figure is divided in two parts. The time evolution during the 22 ns of bias construction is displayed on the lefthand side. The time evolution at constant bias, which constitutes the metastatistics used to compute the biased equilibrium averages, during the last 10 ns is displayed in the righthand side. The figure clearly shows that the unfolding of the protein often occurs during bias construction, since *ξ* decreases from the value characterizing the folded state to values of 3–4 in 3 cases over the 4 displayed. In certain cases (red curve) the expected behaviour of a random walk of *ξ* in the 3–14 range is observed.

In principle metadynamics is capable of letting the system, starting from the known folded configuration, to unfold and refold. In practice this rarely occurs in affordable computational time, unless the system is sufficiently small. To illustrate the time-scale required for collecting such trajectory, the behaviour of *ξ* for the longer FXN chain is displayed in Figure 2 (right panel) for a single walker. In this trajectory a single-walker well-tempered metadynamics is performed for 260 ns. While the first 100 ns of the trajectory displays an ideal behaviour for metadynamics [see for instance Figure 2 in Ref. (Barducci et al., 2006)], when the bias is no more effectively updated by new Gaussian functions the system becomes frozen in a fixed configuration. This effect is expected in well-tempered metadynamics, since the height of the Gaussian functions that are added to the bias is progressively decreased by construction.

Anyway, the dynamics of *ξ* shows that in order to observe a proper random walk of *ξ* for all walkers, simulation time should have been at least 10–100 times larger. The dynamics of *ξ* becomes even slower when the bias is kept constant compared to bias construction (righthand side of both panels in Figure 2). This behaviour is due to the effect of noise during bias construction, occurring when new Gaussian contributions are added to *V*_*G*_ every 10 ps. The dynamical nature of *V*_*G*_ during its construction acts as a stochastic perturbation. This effect is not present when *V*_*G*_ is kept constant and when *V*_*G*_ does not change because added Gaussian heights are small.

Because of the slow dynamics of *ξ*, the metastatistics represents a static disorder triggered by the bias construction process.

**FIGURE 3:**
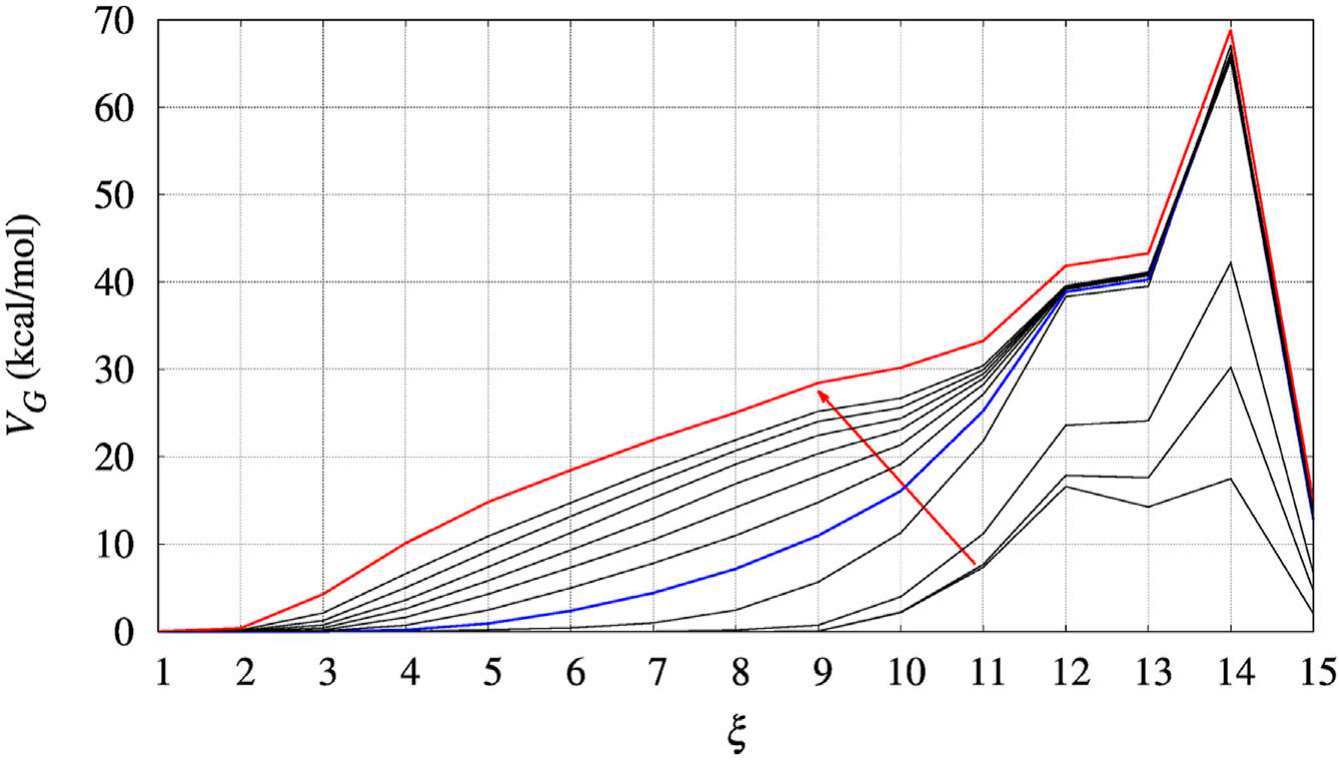
The evolution of the bias (*V*_*G*_) acting on walker 1 during bias construction for BPTI, [5-55]_BPTI_ sequence. Different curves are obtained combining the bias of all walkers using Eq. 3 of Ref. (Hošek et al., 2017). The red arrow indicates the increasing iteration. The red curve is that used in the final collection, while the blue curve was used to estimate the effect of a non-converged bias on the values of free energy obtained by the post-processing MEC method.

In Figure 3 we display the behaviour of *V*_*G*_(*ξ*) during the bias construction for the BPTI reference sequence. The red arrow indicates the direction in which the iteration index of the altruistic method, occurring every 2 ns of metadynamics simulation, increases. In the process of iteration the number of Gaussian contributions to *V*_*G*_ keeps increasing in the region of low *ξ* values, while at high *ξ* it does not change anymore after the first 4 iterations. A full convergence of *V*_*G*_ is not achieved, but we notice that the change of *V*_*G*_ is very slow after about 10 iterations. This happens because when the protein is unfolded, many atomic configurations consistent with a low number of hydrogen bonds are possible. Then, Gaussian contributions to *V*_*G*_ are all added in the region of low *ξ* values, while no further contributions are added to the region of high *ξ* values.

Since the bias converges very slowly, it is worth checking the effect of choosing different bias in the calculation of interest for us, that is, ΔΔ*G* as a function of the chosen collective variable for a protein variant with respet to the wild-type sequence. In Figure 3 we choose the red curve, as what we assumed as converged bias, and the blue curve, the function built after 5 iterations in the altruistic scheme (stage 9 in Table 3). The difference between results obtained with these two different choices of final bias will be described later for BPTI. We remark that the configurations used in the comparison are the results of two different 10-ns trajectories for all of the 90 walkers: one performed with the “converged” bias (stage 15 in Table 3) while the second performed with the bias of stage 9.

Times of the order of 100 ns are required to build a useful bias for each walker even for a protein of 58 residues like BPTI. This issue is illustrated by the behaviour of a single walker of the larger FXN protein (see Figure 2, right panel, discussed above). In practice such long simulation times can not be used to compare a native sequence with the usually rather large number of its variants. The method described in this article allows extracting differences in stability under point variations with computational wall-times of the order of 1 month in a high-performance computing infrastructure.

**FIGURE 4:**
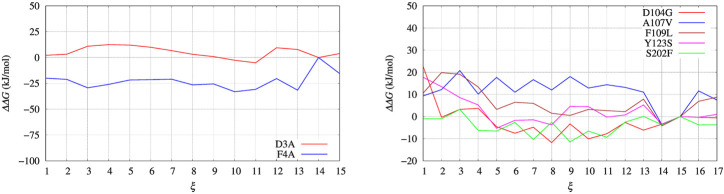
Free energy change (ΔΔ*G*) calculated with Eq. 9 and *V*_*G*_ built with well tempered metadynamics. Left panel: BPTI; Right panel: FXN.

**FIGURE 5:**
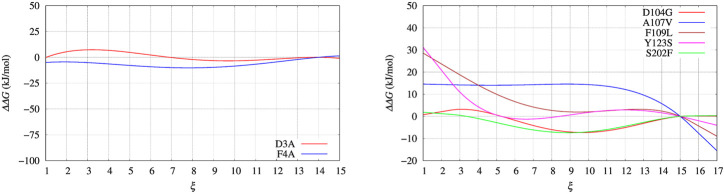
Same as for Figure 4 using the polynomial of order 4 interpolating the grid representation of the bias *V*_*G*_ used in Figure 4. Left panel: BPTI; Right panel: FXN.

**FIGURE 6:**
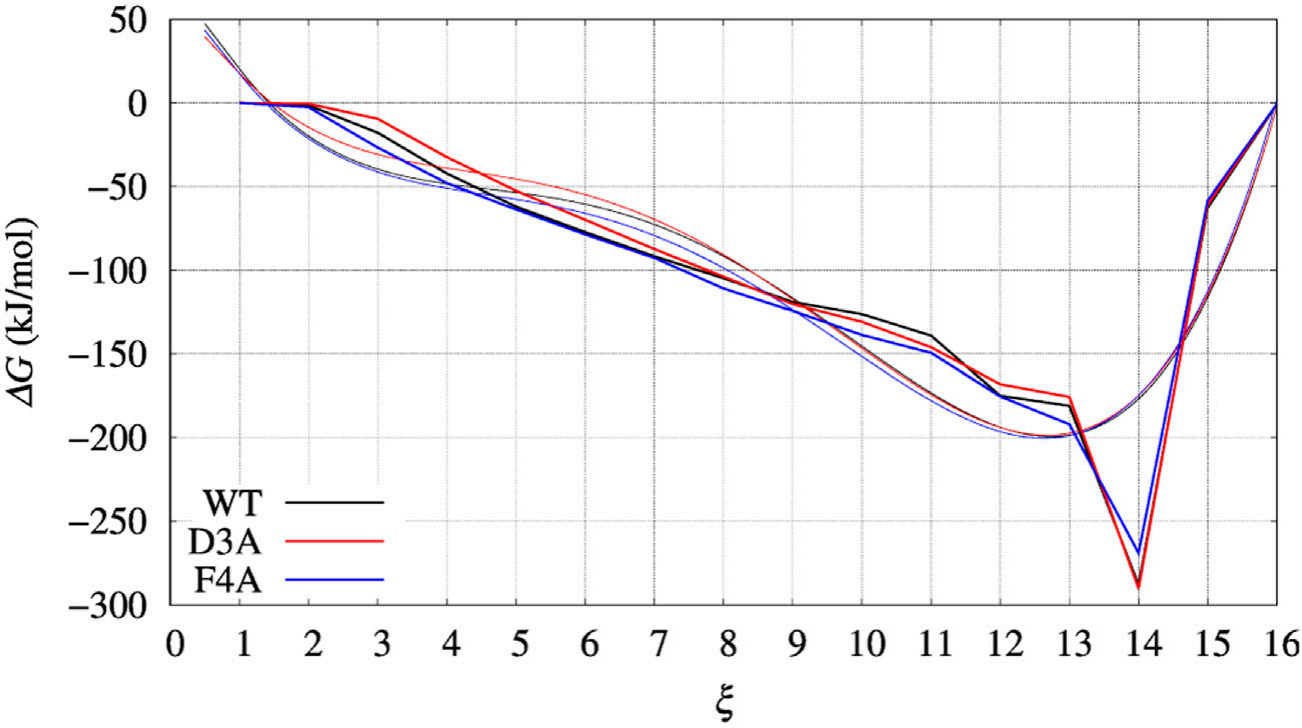
Same as for Figures 4, 5 (left panels), but comparing Δ*G*(*ξ*) for the case of BPTI variants. Color scheme is black, red, and blue for WT, D3A, and F4A variants, respectively. Thick line is the grid representation of *V*_*G*_; thin line is the 4-th order polynomial interpolation of each grid (same color) in the range between 0.5 and 15.5. Out of this range the interpolation is linear, with continuous derivative at the extreme points.

In Figure 4, left panel, we display the free energy change ΔΔ*G* computed using Eq. 9, implicitly assuming that *V*_*G*_ has properly converged after 22 ns of multiple-walkers bias construction. In Figure 5, we also display the free energy change using the polynomial of order 4 interpolating the grid representation of the bias *V*_*G*_ (see Section 2.4). We remind that the polynomial interpolation is performed on each approximately converged *V*_*G*_(*ξ*) profile obtained by metadynamics. Therefore, the effect of interpolation on the free energy change ΔΔ*G* as a function of sequence change can be slightly different when the difference between interpolated curves is extracted. In Figure 6 we display the comparison between the grid representation of Δ*G* = −*V*_*G*_ + *C* and its interpolation in all of the three sequences investigated for BPTI. We notice that for the three variants the free energy increases by decreasing the value of the collective variable, consistently with a greater thermal stability of the protein configurations in the folded state. But, because of the smoothing of *V*_*G*_ exerted by the interpolation, part of the changes are lost. However, even the tiny difference between the curves Δ*G* = *G*(*ξ*)—*G*(*ξ*_0_) is still a representation of the change in stability upon protein unfolding once the sequence is changed. In the BPTI case, we find that, consistently with experiments (see Table 4), the D3A mutation induces an increase in the stability of the protein (left panel, red curve). The opposite is true for the F4A mutation.

**TABLE 4:** Experimental (Δ*T*_*m*_, °C), experimental ΔΔ*G* Petrosino et al. (2019), and computed values of ΔΔ*G* (kJ/mol) for the selected BPTI and FXN variants. Column 4: the values obtained with metadynamics. Columns 5-7: the maximal constrained entropy method is used with the effective energy for solute-solvent interactions (Eqs 18, 19). Column 5—Data published in previous article Botticelli et al. (2022); column 6—Simulation used in previous article, using the extended *ξ*′ variable in the maximal constrained entropy method; column 7—Well tempered metadynamics, using the extended *ξ*′ variable in the maximal constrained entropy method. Rows are reported in descending order of Δ*T*_*m*_ for each protein. While for BPTI the collective variable *ξ* = *α* + *β* is used both in metadynamics and maximal constrained entropy methods, for FXN *ξ* = *β* is used in metadynamics and the extended variable *ξ*′ = *ξ* + *α* = *β* + *α* is used in the maximal constrained entropy method. *β* is the number of hydrogen bonds in the *β*-sheet; *α* is the number of hydrogen bonds in the *α*-helices (see Methods for details). BPTI: Unfolded state is *s* = 4; Folded state is *s* = 14 (highest peak in the distribution obtained with the meta-statistics, see Figure 7). FXN: Unfolded state is *s*′ =21 [23 for simulation of Ref. (Botticelli et al., 2022)]; Folded state is *s*′ =37.

Variant	Δ*T*_*m*_	ΔΔ*G* (exp.)	ΔΔ*G* (calc.)			
BPTI [5-55]_BPTI_						
D3A	1.4	0.84	12.5	-	-	−2.5
F4A	−21.2	−12.55	−26.0	-	-	−56.2
FXN						
D104G	3.0	0.88	2.5	20.1	16.1	58.4
S202F	−0.3	−0.67	−1.0	−7.3	14.6	2.3
A107V	−3.0	3.35	14.1	−114.2	−89.5	−11.9
F109L	−11.4	−8.74	14.1	−21.3	−32.5	−89.9
Y123S	−14.4	−20.59	4.3	−25.2	29.2	−56.4

As discussed above, statistics severely limits the convergence of *V*_*G*_(*ξ*), since the number of Gaussian contributions to *V*_*G*_ giving an almost flat *ξ* distribution is achieved when all unfolded configurations are sampled, a condition, that is, hardly achieved even with *μ*s-long MD simulations. Employing multiple walkers allows one to sample unfolded and folded configurations in non-infinitely long simulation but each of the walkers is not able to walk from a folded configuration towards an unfolded one and viceversa with a frequency allowing a proper sampling.

**FIGURE 7:**
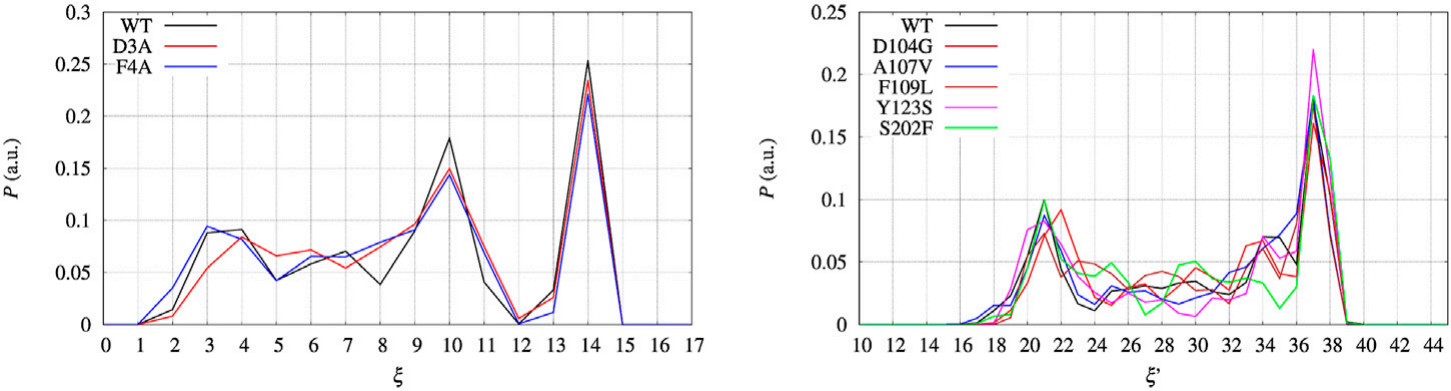
Distribution *P* as a function of the collective variable *ξ*. Left panel—BPTI, where *ξ* = *β* + *α*, where *β* and *α* are the number of hydrogen bonds in, respectively, the *β*-sheet and *α*-helices present in the folded structure. Right panel—FXN, where *ξ* = *β* was used to build the external bias, but the extended variable *ξ*′ = *β* + *α* is used to represent the distribution. *P* is normalized as to have *∑*_*i*_ *P*_*i*_ = 1, where *P*_*i*_ is each of the displayed values.

In the left panel of Figure 7 we display the distribution of the collective variable *ξ* along with the sampling at constant bias *V*_*G*_(*ξ*) for all the BPTI variants. The distributions that we obtain are not flat because of the technical limitations of the well tempered metadynamics method (see Section 2.2) and the limited span of sampled CV values as shown in Figure 2 and discussed above. Despite the sampling being likely insufficient to have both a converged bias and a converged distribution of *ξ* once a constant bias is applied, we can estimate the reversible work necessary to build a given average of *ξ*, *s*, from the biased metastatistics at our disposal. This is done using the maximal constrained entropy approach described in Ref. (Botticelli et al., 2022) and references therein.

**FIGURE 8:**
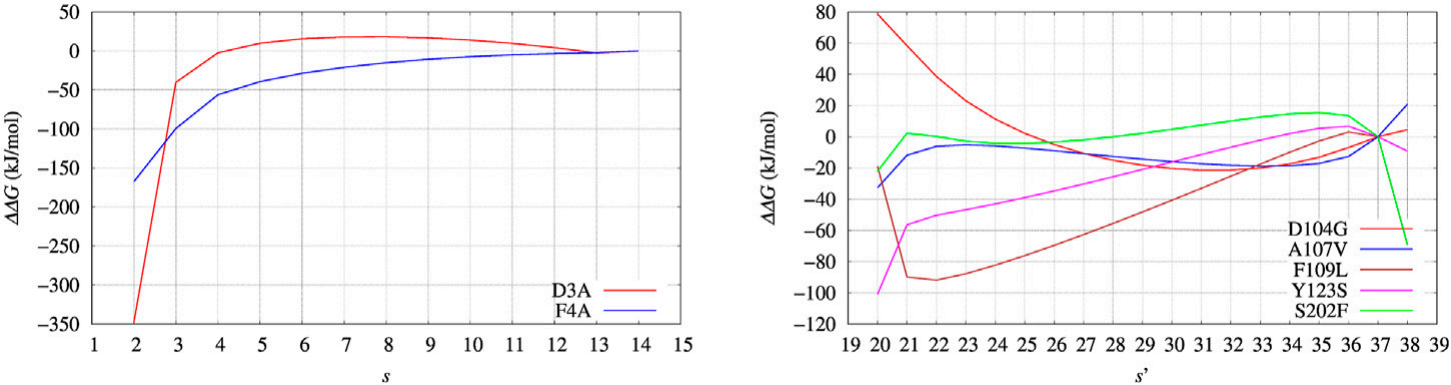
Changes of free energy variation (ΔΔ*G*) upon unfolding, that is, the decrease of the average number of hydrogen bonds in *α* helices and *β*-sheets, *α* and *β*, respectively. The average is *s* = ⟨(*α* + *β*)⟩. As for FXN (right panel) the number of hydrogen bonds is calculated after using metadynamics based on *ξ* = *β* and *ξ*′ = *α* + *β* in the maximal constrained entropy method. Color scheme is the same as for Figure 7.

The free energy difference between each of the two variants of BPTI D3A and F4A, and the reference sequence [5-55]_BPTI_ is displayed in the left panel of Figure 8 as a function of the average value *s* of the collective variable *ξ* (see Section 2). The MEC modulation is employed here and Eq. 1 is used, with *X* sequences identified by different colors. Since the distribution of *ξ* in the metastatistics displays a sharp peak in the folded state (*ξ* = 14) and a broad peak in the unfolded one (at about *ξ* ∼ 4) we report in Table 4 the free energy change going from the state of average *s* = 14 (that is, the state *s*_0_ in Eq. 1) to the state with average *s* = 4.

The direct metadynamics calculation and the maximal constrained entropy method give consistent results for the sign of ΔΔ*G* in the case of BPTI. In fact, we find a slightly negative value equal to −2.5 kJ/mol for D3A at *s* = 4 (see Table 4; Figure 8, left panel). Moreover, in both cases our results are consistent with experiments. It is important to recall that among the 48 single-point mutations of [5-55]_BPTI_, only 3 produces a stabilization of the protein Yu et al. (1995).

**FIGURE 9:**
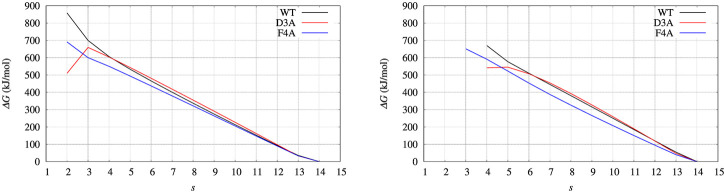
BPTI: change of free energy (Δ*G*) upon unfolding, that is, the decrease of the average number of hydrogen bonds in *α* helices and *β*-sheets, *α* and *β*, respectively. The average is *s* = ⟨(*α* + *β*)⟩. Left panel is obtained with Eqs 10, 18, using the configurations collected for 10 ns with the final bias obtained (red curve in Figure 3); Right panel is obtained using the configurations collected for 10 ns with an intermediate bias, blue curve in Figure 3. Color scheme is the same as for Figure 7.

Before entering into structural details providing explanation of ΔΔ*G* values, we assess what we expect to be the major source of error propagation in the MEC method. The most efficient handle to expand the sampling of atomic protein configurations is the external bias *V*_*G*_, as computed by well-tempered altruistic multiple-walkers metadynamics. Therefore, we calculated Δ*G*(*s*) profiles for BPTI, which is protein small enough to easily repeat 900 ns of MD simulations, using two different choices of *V*_*G*_(*ξ*), respectively the red and blue curves in Figure 3. The profiles of Δ*G*(*s*) computed with the different choices are displayed in Figure 9, left and right panels, respectively, for red and blue curves of *V*_*G*_(*ξ*). We notice that there are a few values that are affected by the limited number of points in the statistics: *s* = 2 (left panel) and *s* = 4 (right panel). By using the bias obtained by a shorter cumulative history (the blue curve), low values of *ξ* (contributing to low values of average *s*) are rarely sampled. Apart from these limitations due to the range of sampled *ξ* values, the similarity in the behaviour of *G*(*s*) is remarkable. In particular, we notice that the sign of ΔΔ*G* (the difference between curves in each of the plots) is robust. This depends on the fact that the contribution to the calculation of Δ*G*s with the help of Eq. 10 depends on the energy of the populated states (with a certain value of the collective variable *ξ*) rather then to the number of ways the state is reached by the simulation.

Due to the collection of atomic configurations at hand and to the possibility of computing the different terms contributing to ΔΔ*G*, we can interpret the unusual stabilization of the D3A variant observed in experiments. The increase in unfolding free energy upon D3A mutation is partially due to the removal of the salt-bridges formed by Asp 3 that occur in the WT sequence. On the other hand, the F4A mutation reduces the steric hindrance of Phe 4 thus enhancing the chance of salt-bridges formation between the N-terminus and other protein regions. The competition between electrostatic long-range contributions and short-range interactions characterizing the hydrophobic patches can be analyzed studying the changes in the terms contributing to *U*.

In Table 5 the change in four terms contributing to *U* (see Eqs 18, 19) are reported, together with the whole change of *U* (last column). The latter dominates the change of *G*, since the contribution of the maximized cross-entropy is small compared to Δ*U* in Eq. 10. The significant changes in each component almost cancel each other in the sum. The smaller change in *U*_*vdw*_ in the F4A case indicates the cancellation of hydrophobic contacts between Phe 4 and the residues in the major hydrophobic core of folded BPTI when Phe 4 is replaced by Ala. The negative change of *U*_*solv*,*np*_ for all variants indicates the release of hydrophilic sidechains into the solvent upon unfolding. This contribution tends to cancel the release of dispersive solute-solute interactions. However, the two electrostatic contributions (Δ*U*_*el*_ and Δ*G*_*solv*,*pol*_) span the largest range of values among the variants. Therefore, we argue that changes in the electrostatic networks become critical with respect to an almost uniform background of interactions that change upon the demolition of the hydrophobic core occurring during unfolding.

**TABLE 5:** Energy changes (kJ/mol) starting from folded reference state, ending to unfolded state for native (WT) sequence and studied variants. Folded and unfolded states are defined as in Table 4. The energy components are those indicated in Eqs 18, 19.

Variant	Δ*U*_*el*_	Δ*U*_*vdw*_	Δ*G*_*solv*,*pol*_	Δ*G*_*solv*,*np*_	Δ*U*
BPTI [5-55]_BPTI_					
WT	749.8	198.3	−264.1	−47.8	608.0
D3A	802.7	196.2	−294.4	−58.4	605.1
F4A	660.2	159.5	−170.0	−58.7	552.4
FXN					
WT	910.8	542.7	−56.0	−127.9	1218.0
D104G	1295.0	544.9	−394.6	−133.2	1275.8
A107V	986.7	553.0	−125.1	−146.6	1206.3
F109L	788.9	583.8	−33.9	−134.3	1127.7
Y123S	754.4	563.2	37.0	−138.4	1161.3
S202F	911.1	577.0	−65.4	−135.9	1219.9

Most of the long-range salt-bridges lock the native structure into a less hydrophilic globular form, because the small size of the globule allows efficient electrostatic sealing, not allowed when the size of the globule increases. Breaking of the salt-bridges in the native form allows exposing hydrophylic groups to the solvent while the formation of the salt-bridges hides hydrophobic groups inside the globule core. Once salt-bridges are broken, that is, when the hydrogen bonds keeping the native scaffold are broken, the globular protein is allowed to expose a larger surface to the solvent, including its hydrophobic core.

In conclusion, the D3A stabilization against protein unfolding is due to the stabilization of non-native salt-bridges when the native Asp 3 is removed.

Our analysis has shown that for the small BPTI protein (58 residues) the number of configurations at constant bias we have been able to collect provides consistency between well tempered metadynamics and the maximal constrained entropy method. On the other hand, the improvement of statistics we achieve in this work, compared to our previous investigation of the FXN case, as explained in the next section, is not yet sufficient to get full consistency and robust predictions in the case of bigger proteins.

### 3.2 Frataxin (FXN)

The effects of single-point mutations on the unfolding process of the truncated form of FXN (residues 90-210) have been discussed in detail in Ref. (Botticelli et al., 2022). Differences of the present work compared to what was done in the previous paper are the following:

1. The well tempered metadynamics method is employed in place of a plain (constant *T*) metadynamics;
2. The construction of the biasing potential is made with a larger number of iterations and is, therefore, more accurate;
3. The maximal constrained entropy method is applied here using an extended collective variable including the number of hydrogen bonds present in the folded *α*-helical regions;
4. The trajectory produced at constant bias, which is used in the maximal constrained entropy method, is three times longer than in Ref. (Botticelli et al., 2022).

The change of *G* computed using the bias *V*_*G*_(*ξ*) as obtained out of 22 ns of bias construction is displayed in the right panels of Figures 4, 5. Again, the free energy increases upon unfolding (decrease of *ξ*), in agreement with what happens in similarly folded state as observed in experiments Petrosino et al. (2019) (data not shown here). However, the relative order of unfolding free energy is not well captured. Indeed, most of the variants are found to be more stable than the WT reference sequence. On the other hand, in experiments only the D104G variant among the 8 analyzed shows an increased stability of the folded state with respect to the native sequence and, therefore, a larger unfolding free energy.

**FIGURE 10:**
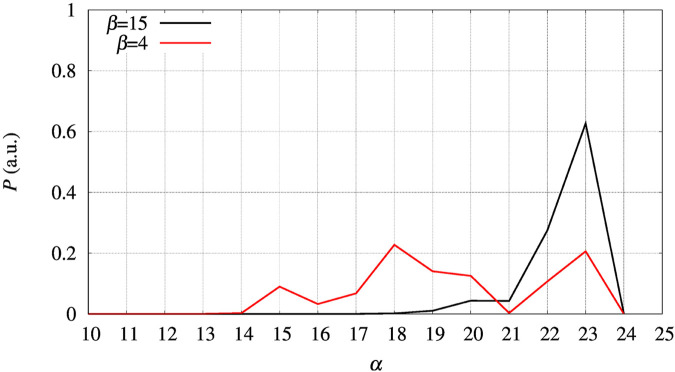
The distribution of *α* obtained in the 30-ns trajectories collected for the whole set of 90 walkers at constant bias *V*_*g*_(*β*) for FXN, WT sequence. Black curve—Distribution obtained for configurations with *β* = 15; red curve—Same distribution with *β* = 4.

An explanation of the difference between the trend showed by experiments and that predicted by direct metadynamics is in the choice of the collective variable we made to study FXN unfolding. The thermal unfolding was measured by CD at 222 nm wave-length: this means that the CD signal was mainly composed by variations in the content of *α*-helices. The choice of *ξ* = *β* in metadynamics was based on the expectation that the demolition of the *β*-sheet would be sufficient to destabilize all the secondary motifs in the protein, including the two *α*-helices. This was only partially true. In Figure 10 we show the distribution of *α*, the number of hydrogen bonds in *α*-helices, in correspondence low and high values of *β*, 4 and 15, respectively. The distributions were computed making reference to the 30-ns long simulation at constant bias collected for the whole set of 90 walkers. The curve with *β* = 4 shows that *α*-helices are partially broken in those configurations where the *β*-sheet is broken. This effect is due mainly to the shortening of helix *α*_1_ (data not shown here), which is softer than *α*_2_ particularly in its N-terminus. Therefore, only an *a posteriori* analysis of the effect of a chosen collective variable can point to a more valid collective variable to be used in metadynamics.

The set of configurations, obtained by including all the 90 walkers simulated at constant bias, is used in the maximal constrained entropy method to overcome the above shortcoming. Results for FXN are displayed in the right panels of Figures 7, 8. In Figure 7 (right panel) we notice that the two peaks at *s*′ = 21 and 37 are not due just to the choice of initial configurations (i.e. the two PDB structures used to differentiate the walkers, see Section 2). The distance in *s*′ between the two peaks displayed in Figure 7 (right panel) is larger than the difference in *α*-helical values between the two PDB structures used to build the set of initial configurations, namely 37-21 compared to 23-19. Consistently with the data displayed in Figure 10, this means that the metastatistics contains configurations with a significant decrease in the number of *α*-helical hydrogen bonds despite the external bias forcing the unfolding being a function of the number of hydrogen bonds in the *β*-sheet only.

In the right panel of Figure 8 the profiles of ΔΔ*G* of the 5 different variants studied with maximal constrained entropy method are compared. It is interesting to notice that the relative order of the experimental values of Δ*T*_*m*_ (see also Table 4) is better reproduced with the use of the augmented and updated statistics collected in this work.

The different contributions to ΔΔ*G* are reported in Table 5. Again, the tendency of different contributions to compensate each other when summed is apparent. It can be noticed that, similarly to BPTI, the electrostatic contributions display a larger span among variants. In the case of D104G the value of Δ*U*_*el*_ is clearly dominant, while the opposite sign contribution of the polar solvation term is unable to compensate the effect of changes in direct electrostatic contacts. Strikingly, despite the longer accumulation of statistics and the more accurate bias construction, the reasons of the D104G stabilization can be explaind in terms of the same effects described in the previous investigation Botticelli et al. (2022). It is the removal of Asp 104 that changes the structure of the *α*_1_ helix and the possibility of the charged residues lying in that region to form alternative salt-bridges. When *α*_1_ helix is allowed to rotate, like in the unfolded molten globule, these interactions are not possible. However, the effect of the point mutation on the S202F variant is different as the change of dispersive interactions become significant, consistently with the introduction of a hydrophobic sidechain (Phe) in place of the small hydrophilic Ser residue. In this situation, it is possible to infer that the native-like hydrophobic core is stabilized and more work is required to destroy it and the significant change in electrostatic interactions (Δ*U*_*el*_) is seen to positively combine with hydrophobic contributions.

Though the interactions among protein atoms and between the protein and its environment (a NaCl solution) are crudely approximated, the method is able to capture the little changes surviving when the total potential energy is computed.

## 4 Conclusion

In this work we refined the combination of several computational methods to predict, on the basis of fully atomistic protein models, the changes of thermal stability of proteins under single-point mutations. The method has been applied to a well-studied small protein, the bovine pancreatic trypsin inhibitor (58 residues), and to a truncated form of frataxin (121 residues). In both cases experiments were compared to computational results. The unusual effect of protein stabilization exerted by some point mutations was the special focus of this study.

We found a good agreement in the sign of representative values of ΔΔ*G* upon unfolding and the sign of the shift in the melting temperature compared to experimental results. The competition between the changes in the demolition of hydrophobic cores and the changes in networks of electrostatic interactions is captured by the method. This effect was not fully analyzed in the interpretation of the unusual D3A stability in BPTI, so far.

Despite its potential, the method is computationally quite demanding, requiring extended statistical methods and, as for the collection of reliable configurations, a detailed model for atomic interactions, including explicit solvent and counterions. As discussed in the case of FXN, the direct calculation of free energy variation from the constructed bias potential is strongly affected by the choice of the collective variable in metadynamics. It was found that the maximal constrained entropy is a possible work-around to the statistical limitations of even challenging and promising methods like those based on multiple-walkers well tempered metadynamics. Numerical limitations still prevent the application to many interesting variants where the native structure becomes unstable: F33A, F22A, Y35A for BPTI; W173C for FXN. The ability of predicting the sign of the free energy change is in any case of extreme importance when the protein can adopt structures alternative to the native one.

## Data Availability

The raw data supporting the conclusion of this article will be made available by the authors, without undue reservation.
